# Von der Philosophie‐ zur Sozial‐ & Wirtschaftsgeschichte. Zwei Varianten des Exzerpierens beim jungen Marx

**DOI:** 10.1002/bewi.201900027

**Published:** 2020-05-14

**Authors:** Axel Rüdiger, Harald Bluhm

**Affiliations:** ^1^ Martin-Luther-Universität Halle-Wittenberg Institut für Politikwissenschaft Abderhalden Str. 26 Halle 06099 Germany; ^2^ Martin-Luther-Universität Halle-Wittenberg Institut für Politikwissenschaft Abderhalden Str. 26 Halle 06099 Germany

**Keywords:** Karl Marx, Exzerpieren, Philosophiegeschichte, Wirtschaftsgeschichte, Sozialgeschichte

## Abstract

This article investigates forms of excerpting and their variations as used by Marx. It compares two convolutes of excerpts from the early period of Marx’ work. The first form of excerpting is represented by the “Hefte zur epikureischen Philosophie” (1839/40), which Marx originally created for his dissertation. These booklets aim to reconstruct the history of post‐Aristotelian classical philosophy, with Epicurus at its centre, from fragments and testimonies of third authors, which themselves are often excerpts from excerpts. A second variant is represented by the so‐called Gülich‐excerpts (1846/47), an extensive source collection of economic history. These excerpts later served as a comprehensive and easily accessible basis for the *Contribution to the Critique of Political Economy* that Marx was already planning at that time. Both variants of excerpts are analysed with regard to the source‐critical method and the acquisition of knowledge in the situation of the new formation of philosophical, social and economic history. This allows for the in‐depth examination of the self‐reflexive practice of excerpting and to shed light on the constitutive tension between copying, critical selection, commentary and autonomous text production.

## 1 Einleitung

Die historische Schule hat das Quellenstudium zu ihrem Schiboleth gemacht, sie hat ihre Quellenliebhaberei bis zu dem Extrem gesteigert, daß sie dem Schiffer anmuthet, nicht auf dem Strome, sondern auf seiner Quelle zu fahren.^1^

2

Die umfangreichen Exzerpte von Karl Marx (1818–1883) werden nach Abschluss der *Marx‐Engels‐Gesamtausgabe* (MEGA^2^) zusammen mit den überlieferten Notizen und Marginalien allein 32 Bände umfassen, präsentiert in einer eigenen IV. Abteilung des Editionsprojektes (worin bisher bereits 14 Bände erschienen sind). Werden pro Band – in einer groben Schätzung abzüglich der Exzerpte von Friedrich Engels und der editorischen Kommentare – nur ca. 300 Druckseiten für Marx reserviert (was eher untertrieben ist), dann ergibt sich hieraus ein Archiv von insgesamt immerhin 10.240 Seiten! Hinzu kommen die hybriden ökonomischen Manuskripte, die teilweise auch ausführliche Exzerpte enthalten, aber aufgrund ihres ökonomischen Zusammenhanges in der Zweiten Abteilung ediert wurden, die dem Hauptwerk, dem *Kapital*, gewidmet ist. Schon angesichts der üppigen Menge wird klar, dass das Exzerpieren für die wissenschaftliche Arbeit von Marx hohen Stellenwert besitzt. Mehr noch: Seine Exzerpte eignen sich als repräsentatives Beispiel für die anhaltende Relevanz dieser gelehrten Praxis im 19. Jahrhundert, was einschließt, dass diese Praxis keineswegs verschwindet, sondern seine Methoden, Formen und Diskurse bis in die Gegenwart hinein immer wieder neu erfindet und weiter entwickelt.^2^

Wie das Motto von Marx aus dem Jahre 1842 anzeigt, ist seine Exzerpierpraxis im Zusammenhang der philologischen Quellenkritik schon früh diskursiv und reflexiv vermittelt. Die Kritik am Quellenfetischismus der historischen Rechtsschule enthält unserer Ansicht nach eine symptomatische Abgrenzung von älteren Formen rein rezeptiven Buchwissens, das im Kontext der Debatten um die Französische Revolution und der ihr vorausgegangenen Aufklärung für Marx im deutschen Vormärz einen konservativen Eindruck machte. So zog er aus der akademisch‐politischen Fehde seiner Lehrer Eduard Gans (1797–1839) und Friedrich Carl von Savigny (1779–1861) an der Juristischen Fakultät in Berlin, in der es um den historisch‐philologischen oder rational‐politischen Charakter des Rechtes ging,^3^ offenbar die Schlussfolgerung einer kreativen Synthese aus philosophischer Autonomie im rationalen Modus des Selbstdenkens und dem intensiven Studium historischer Literatur, wobei die individuelle, historische Quelle den allgemeinen „Strom“ des rationalen Denkens allerdings zu speisen und keinesfalls als „Schiboleth“ zu ersetzen habe. Dass Marx bei dieser rationalen Rekonfiguration des gelehrten Buchwissens seinen Meister Hegel zu übertreffen suchte, indem er nicht nur theoretisch die Absicht verfolgte, auf die Schultern dieses philosophischen Riesen zu klettern, sondern dies auch ganz praktisch in seiner konkreten Exzerptarbeit umsetzte, wollen wir stichprobenartig anhand der 1839/40 und 1846/47 angefertigten Exzerpthefte zur epikureischen Philosophie und zur globalen Wirtschaftsgeschichte typologisch demonstrieren. Bisher ist der Umstand, dass das Exzerpieren die Basis für den im Aufbau befindlichen theoretischen Überbau bildet, die erst zusammengenommen die gelehrte Produktionsweise von Marx ausmachen, selten berücksichtigt worden.^4^

Wir wenden uns vor diesem Hintergrund mit der von Décultot und Zedelmaier entwickelten Heuristik einem relativ frühen Stadium von Marxens Schaffen zu, in dem er seine Arbeitsweise in zwei Varianten des Exzerpierens ausprägt. Neben der uns leitenden Fragestellung nach dem Zusammenhang von Exzerpieren und Autorschaft interessiert uns das Verhältnis von Lektüre und Empirie näher, weshalb uns der Vergleich des philosophiehistorischen mit einem wirtschafts‐ und sozialwissenschaftlichen Exzerptkonvolut besonders geeignet erscheint. Lässt sich in der Exzerpierpraxis von Marx tatsächlich etwas Neues feststellen, das sich sowohl vom älteren Bücherwissen abhebt als auch den methodischen Historismus der Rechtsschule sowie die weltgeschichtlich abstrakte Perspektive Hegels synthetisierend übersteigt? Wir fragen zudem, ob es Leitgedanken des älteren Exzerpierdiskurses gibt, die tradiert werden? Welche Rolle spielt die Urteilskraft des Autors bei der konkreten Form des Exzerpierens, etwa bezüglich der Unterscheidung von bloßer Kopie und kritischer Auswahl geeigneter Textpassagen und Ideen? Gibt es folglich unterschiedliche Methoden, die über das einfache Notieren von Stellen hinausgehen? Wird resümierend oder polemisierend kommentiert? Ferner interessiert uns die Ordnung der Exzerpte, ihr räumlich‐materieller Aspekt ebenso wie die verwendete Systematik – gab es neben topisch geordneten Heften weitere Repositorien? Auf diese Weise wollen wir zu einem differenzierteren Verständnis von Marx’ früher Exzerpiertätigkeit hinsichtlich ihrer sachlichen und fachlichen Modalitäten im historischen Kontext gelangen. Am Beginn steht die Klärung, welche Texteditionen Marx beim Exzerpieren nutzt.

## 3 Der „Copist“ als „Maulwurf“: Die „Hefte zur epikureischen Philosophie“ von 1839/40

Marx hatte Ende 1838 beschlossen, anstatt in seinem eigentlichen Studienfach der Jurisprudenz in der Philosophie zu promovieren.^5^ Diese berufspraktisch durchaus riskante Wahl dürfte von seiner damaligen engen Verbindung zu Bruno Bauer (1809–1882) und dem philosophischen Gesprächskreis der Junghegelianer inspiriert gewesen sein. Inhaltlich plante er eine Arbeit im Bereich der antiken postaristotelischen Philosophiegeschichte. Die junghegelianische Interpretation von Epikureismus, Stoizismus und Skeptizismus versuchte Hegels Deutung dieses nacharistotelischen Denkens im Paradigma des Selbstbewusstseins auf das hegelsche System anzuwenden, um auf diese Weise eine selbständige philosophische Position zu entwickeln.^6^ Insofern ging es darum, aus dem großen Schatten Hegels herauszutreten und eine eigene philosophische Position zu artikulieren, die an den Grundsätzen des hegelianischen Systems zwar prinzipiell festhielt, aber sich vom Eindruck des bloßen Epigonentums emanzipierte. Nachdem 1836 der dritte und letzte Band von Hegels *Vorlesungen zur Geschichte der Philosophie* veröffentlicht worden war, hatte Bauer 1838 in seiner *Kritik der Geschichte der Offenbarung* erste Ansätze für ein entsprechendes Theorieprogramm vorgelegt, in dem sich die seit der Frühaufklärung gestellte Frage nach dem Verhältnis von Offenbarungsreligion und Philosophiegeschichte zu wiederholen schien. Davon motiviert begann Marx Anfang 1839 mit dem Studium der Philosophie Epikurs, in dessen Verlauf er sieben Exzerpthefte anlegte.

Die sieben Hefte wurden von Hand aus festem weißem Papier angefertigt, wobei die einzelnen Seiten durch Fäden zusammengeheftet sind.^7^ Die Formate der Papierbögen betragen bei Heft 1 gefaltet 288×186 Millimeter und 284×192 Millimeter bei den übrigen Heften. Der Umfang der Hefte schwankt zwischen 22 Seiten (Heft 1), 24 Seiten (Heft 2–5), 16 Seiten (Heft 6) und 12 Seiten (Heft 7). Die einzelnen Seiten sind sowohl ein‐ als auch zweispaltig beschrieben, wobei die letzteren besonders eng beschriftet sind. Die Hefte enthalten leere Seiten und am Ende von Heft 1, 2 und 4 Textteile, die aus dem kontinuierlichen Arbeitsfluss herausfallen und in einer späteren Bearbeitungsphase nachgetragen wurden. In der MEGA^2^ sind die Exzerpte unter dem Titel „Hefte zur epikureischen Philosophie“ vollständig auf 136 Druckseiten ediert, wobei die später hinzugefügten Textbausteine unter dem gesonderten Titel „Fragmente von Epikur‐Studien“ (4 Seiten) abgedruckt sind.^8^

Da die einzigen überlieferten Originalfragmente Epikurs damals editorisch noch nicht erschlossen waren und somit keine ursprünglichen Texte vorlagen, zudem auch noch keine Sammlung der indirekten Wiedergabe existierte, musste Marx seine Quellen aus den verfügbaren Editionen der antiken Literatur exzerpierend zusammentragen.^9^ Die Tatsache, dass er hierbei nicht auf Jacob Bruckers *Historia critica* (1742–1744) zurückgriff, die im 18. Jahrhundert höchste philosophiegeschichtliche Autorität genoss, und aus der neben den französischen Enzyklopädisten auch noch Kant und Hegel großzügig geschöpft hatten,^10^ lässt darauf schließen, dass Marx dem Prinzip *ad fontes* Vorrang vor schneller Generierung von Textmaterial aus zweiter oder dritter Hand einräumte. Mehr noch als die Großmeister versuchte er den historischen Quellen so nahe wie möglich zu kommen und stellte Immanuel Kants „philosophische Archäologie“ und Hegels spekulative Synthese aus Geschichtsphilosophie und Philosophiegeschichte so von Beginn an auf ein materiales historisches Textfundament.^11^ Damit verbindet sich in der Exzerpierpraxis von Marx die logische mit der historisch‐philologischen Methode in der Philosophiegeschichtsschreibung, wobei die quellenkritische Dimension seines Vorgehens noch dadurch unterstrichen wird, dass er auf originalsprachliche Editionen – zumeist aus dem 16. und 17. Jahrhundert – zurückgreift. Da Marx der quellennahen Arbeitsweise auch später treu bleibt, sprechen wir vom quellenkritischen Exzerpieren als der bevorzugten literarischen Produktionsweise von Marx, differenzieren diese aber der Form nach in *kursorisches*, *statarisches*, *disziplin*‐ und *problemgeschichtliches* Exzerpieren. Im Gegensatz zur zusammenfassenden Arbeitsweise bei den kursorischen Auszügen handelt es sich beim statarischen Modus um ein Zeile für Zeile vorgehendes Exzerpieren, das eng mit einem hermeneutischen Lesetypus verbunden ist, der dem Konzept des *close reading* nahekommt.

Marx beginnt mit Diogenes Laertius – dem erklärten Stammvater der eklektischen Philosophiegeschichte – und exzerpiert in den beiden ersten Heften das Epikur betreffende zehnte Buch aus *De clarorum philosophorum vitis, dogmatibus et apophthegmatibus libri decem* im griechischen Original nach einer von Pierre Gassendi besorgten Ausgabe von 1649.^12^ Es folgen im zweiten Heft Auszüge aus Sextus Empiricus und Plutarch. Letztere werden im Heft 3 und 4 fortgesetzt. Das Heft 4 enthält des Weiteren das Exzerpt aus Lukrez, welches im Heft 5 fortgesetzt wird. Anschließend exzerpiert Marx jüngere Sekundärliteratur (Ferdinand Christian Baur und Heinrich Ritter). Hinzu kommen Notate aus Seneca, Ioannes Stobaios und Clemens Alexandrinus im Heft 6 sowie Cicero im Heft 7. Insofern folgt der inhaltliche Aufbau der sieben Exzerpthefte der Abfolge der ausgewerteten Literatur.

Dass Marx mit Diogenes Laertius begann, kann als ein Anschluss an Bauers Offenbarungskritik gedeutet werden, denn Laertius war ein Schlüsselautor für die frühneuzeitliche Philosophiegeschichte, dessen Wiederentdeckung im 16. Jahrhundert im Zusammenhang mit der Rezeption seiner eklektischen Methode schließlich im 18. Jahrhundert zum Ausschluss der Offenbarungsproblematik aus der Philosophiegeschichte führte. Damit büßte die Philosophiegeschichte ihre apologetische Funktion für den religiös gedachten Wahrheitsbegriff ein. Die eklektische Philosophiehistorie sammelt nach dem Vorbild von Laertius die verschiedenen philosophischen Stellungnahmen orientiert an der „gelehrten Historie“ (*historia literaria*) und prüft sie auf ihre philologische Evidenz (*ars critica*), ohne jedoch eine logische Verbindung zu einer metaphysischen Wahrheit im Sinne der religiösen Offenbarung herzustellen.^13^ Allerdings geriet dieser methodische Eklektizismus ebenso wie der Empirismus der Aufklärungsphilosophie im Rahmen des Deutschen Idealismus unter heftige Kritik, die offenbar auch noch von Marx geteilt wurde, was sich an seiner durchweg pejorativen Verwendung des Eklektizismusbegriffs ablesen lässt. Diese deutet darauf hin, dass seine eigentümliche Kombination von logischer und historischer Methode keinen historistischen Rückfall in den älteren Eklektizismus zuließ. Wenn der junge Marx zu dieser Variante des gelehrten Buchwissens auf Abstand geht, stellt sich die Frage, was dies für seine konkrete Exzerpierpraxis bedeutet. Insofern sind die „Hefte zur epikureischen Philosophie“ ein dankbarer Untersuchungsgegenstand, da gerade hier die disziplingeschichtliche Variante des Exzerpierens beim philosophiehistorischen Vorgehen sich mit der problemgeschichtlichen Exzerpierform überschneidet, die danach trachtet, logisch‐philosophische und historisch‐literarische Argumente auf neue Weise miteinander zu kombinieren.

Was lässt sich aus dieser Perspektive über die Praktiken des Exzerpierens sagen? Marx fertigt auf den ersten Seiten seines Exzerptes durchweg griechische Auszüge in Form von wörtlichen Zitaten an, die lediglich durch einige Seitenanstreichungen hervorgehoben werden. Inhaltlich interessiert er sich dabei für Epikurs Version der auf Demokrit zurückgehenden antiken Atomlehre in der Naturphilosophie. Dabei dominiert zunächst das kursorische Exzerpt. Nach einer längeren Phase rezeptiven und sachorientierten Exzerpierens setzen dann eigene schriftliche Reflexionen ein. Der erste eigenständige Kommentar gilt „Epikur als der Philosoph der Vorstellung“, der das Bewusstsein am „consequentesten“ in der Vorstellung verankert habe, so dass er „ebenso wie die Skeptiker von der andern Seite die alte Philosophie [vollendet].“^14^ Sachlich geht es Marx vor allem darum, Epikur und die Skeptiker in eine Kontinuität mit der aristotelischen Philosophie zu rücken. „Die epikureische Philosophie ist wichtig wegen der Naivetät, mit welcher die Consequenzen ausgesprochen werden ohne die moderne Befangenheit.“^15^

Im zweiten Exzerptheft setzt Marx zunächst die Auswertung von Laertius fort, wobei sich Zitate und Kommentare nun quantitativ die Waage halten. Hierbei lässt sich schon archetypisch die später perfektionierte Arbeitsweise von Marx beobachten, bei der die schriftliche Aneignung von Wissen über Exzerpte und Kommentare direkt in Kritik übergehen, die den Ausgangspunkt für die eigenen Publikationen darstellt. Inhaltlich geht es um das Verhältnis von empirischer und spekulativer Methode in der Naturphilosophie sowie den epikureischen Begriff des „Intermundiums“, der den leeren „Zwischenraum von Welten“ bezeichnet und eine innerweltlich‐natürliche Erklärung des schöpferischen Verhältnisses von Sein und Nichts ermöglicht.^16^ Die Bedeutung, die Marx dieser Textstelle beimisst, zeigt sich auch darin, dass er sie aus dem Griechischen direkt ins Deutsche übersetzt, was bis dahin noch nicht erfolgt war.^17^

Ebenfalls noch im zweiten Arbeitsheft wendet sich Marx den „Pyrrhonischen Hypothesen“ des Skeptikers Sextus Empiricus zu.^18^ Bestimmt werden soll das „Verhältniß der epikuräischen Philosophie zum Skepticismus, soweit sich dieses aus Sextus Empiricus ergiebt.“^19^ Wiederum wird im griechischen Original exzerpiert, wobei die kursorischen Zitate gelegentlich durch eigene Reflexionen unterbrochen werden. Marx widmet sich hauptsächlich der Beziehung von empirischem und apriorischem Wissen bei Epikur und den Skeptikern.^20^ Er notiert sich das gemeinsame kritische Verhältnis gegenüber den „positiven Wissenschaften“, was allerdings aus verschiedenen Motiven gespeist werde. Marx vergleicht diesen Gegensatz zwischen Epikureern und Skeptikern mit dem „Verhältniß, zwischen den Frömmlern und Kantianern in ihrer Stellung zur Philosophie.“^21^ Letztlich stuft er den Epikureismus damit als wesentlich nützlicher für die moderne Geistesphilosophie ein als die antike Skepsis.

Als nächstes studiert Marx die moralphilosophischen Schriften und Dialoge des platonischen Epikur‐Kritikers Plutarch.^22^ Dieses Exzerpt umfasst über den Rest des zweiten Heftes hinaus das gesamte dritte und den Anfang des vierten Heftes.^23^ Im Unterschied zu den vorangegangenen Auszügen enthält es anfänglich jedoch kaum Zitate oder inhaltliche Zusammenfassungen. Der sachliche Exzerpierstil schlägt nun in einen höchst polemischen um, wobei der auslösende Moment der Vorwurf des Vulgärmaterialismus gegen Epikurs Bestimmung des höchsten Gutes bildet. Plutarchs „krasse Auffassung der epikuräischen Philosophie“, die den vorgeblich geistlosen Materialismus Epikurs sarkastisch aus platonischer Perspektive kritisiert, wird auf „plumbe Renomisterei“ und „gänzliche Impotens […] zur philosophischen Kritik“ zurückgeführt.^24^ Marx kehrt die herablassende Polemik Plutarchs komplett um und nutzt schon hier eine polemisch‐scharfzüngige Rhetorik, die für seinen Schreibstil später so typisch werden sollte. Offenbar dient diese frühe Arbeitsphase der exzerpierenden Lektüre als Probebühne, um möglichst kräftige metaphorische Bilder für grundsätzliche Kritik und die Durchsetzung der eigenen Sichtweise zu entwickeln. Marx lotet auf diese Weise die eigene Autorenrolle aus, die er auch später immer wieder mit pointierter Polemik gegenüber anderen Autoren verbindet.

Um Plutarch zu kritisieren, sucht Marx diesem in immer neuen Wendungen jegliche philosophische Satisfaktionsfähigkeit abzusprechen und hierarchisiert als kanonisch geltende Autoren auf eigene Weise. So „faselt“ Plutarch „in Gemeinplätzen“ und „raisonnirt, wie ein Handwerksbursche.“^25^ Anstatt zu philosophieren fabriziere der „Philister“ – ein Schimpfwort, dass Marx mit Novalis, Clemens Brentano und Heinrich Heine teilt^26^ – die „albernsten Pinseleien und Gemeinplätze“, vertrete eine „synkretistische Gedankenlose Manier“, die letztlich auf nichts anderes als eine „immanente, selbstgefällige Dummheit“ hinauslaufe.^27^ Wenn Plutarch dann auch noch vorgeworfen wird, er vereine in seiner Person den „dumme(n) Eklektiker“ mit „platonische(m) Pedantismus“,^28^ dann berührt sich das disziplingeschichtliche mit dem problemgeschichtlichen Exzerpt, insofern die historisch‐empirische und die logische Methode in den Begriffen der vorhegelianischen Philosophiegeschichte des 18. Jahrhunderts aufgerufen werden.^29^ Erst nachdem Marx die philosophische Autorität Plutarchs demontiert hat, beginnt er im dritten Arbeitsheft erneut mit der sach‐ und problemgeschichtlichen Auswertung.

Die anhand von Plutarch entworfene Kritik an der falschen Opposition von eklektischer und platonischer Philosophiegeschichtsschreibung bietet einen Einblick, wie Marx seine eigene Praxis des Exzerpierens mit der an Hegel orientierten Synthese von philosophischer Vernunft und historischer Kontingenz zusammendenkt. Da Marx die eklektische Behandlung der Philosophiegeschichte, in der die interne philosophische Logik lediglich als dummes „exoterisches Faktum“ kompiliert wird,^30^ ebenso brüsk ablehnt wie den „platonischen Pedantismus“, eröffnet sich eine alternative Sichtweise auf die substantielle Rolle, die Vernunft und Weisheit in der Geschichte spielen. Alternativ meint hier, dass die alte christliche Idee einer *translatio sapientae*, deren universale Offenbarungswahrheit sich in der konkreten Geschichte offenbart, mit Hegels Geistphilosophie nicht vollständig abgelehnt wird, wohl aber deren traditionelle christlich‐platonische Form, die eine primordiale „Kontinuität von Philosophie und Offenbarung“ unterstellt.^31^ In diesem Sinne fragt Marx nach der allgemeinen Weisheit als „Bestimmung des σοφóς“, die das „Objekt der epikuräischen, stoischen und skeptischen Philosophie“ bildet, wobei er diese letztlich „am consequentesten“ in der „atomistische[n] Philosophie des Epikur“ ausformuliert findet.^32^

Von hier aus lässt sich eine erhellende methodische Verbindung herstellen zum Cicero‐Exzerpt im siebenten Arbeitsheft, wo Marx die Idee der *translatio sapientiae* ganz hegelianisch mit der Metapher des halbblinden dialektischen Maulwurfs verknüpft. „Die philosophische Geschichtsschreibung“, heißt es dort,

hat […] zu trennen; den stumm fortwirkenden Maulwurf des wirklichen philosophischen Wissens von dem gesprächigen, exoterischen, sich mannigfach gebährdenden phänomenologischen Bewußtsein des Subjekts, das das Gefäß und die Energie jener Entwicklungen ist […].^33^

Erst diese Trennung, die nun die vorher erwähnte Opposition von „platonischem Pedantismus“ und eklektischer Stipulation ersetzt, so fährt er fort, bildet den „*kritische[n] Moment*“, der notwendig ist,

um die wissenschaftliche Darstellung eines Systems mit seiner historischen Existenz zu vermitteln, eine Vermittlung, die nicht zu umgehn ist, eben weil die Existenz eine historische ist, zugleich aber als eine philosophische behauptet, also ihrem Wesen nach entwickelt werden muß.^34^

Dies bedeutet für die philologische Quellenarbeit, dass der exzerpierende Philosophiehistoriker unbedingt „Wesentliches und Unwesentliches, Darstellung und Inhalt“ trennen muss; „er dürfte sonst nur abschreiben, kaum übersetzen, noch weniger dürfte er selbst mitsprechen oder ausstreichen etc. Er wäre bloser Copist einer Copie.“^35^ An dieser Stelle kommt Marx über die verbindende Fragestellung von disziplin‐ und problemgeschichtlichem Exzerpieren direkt auf Fragen zu sprechen, die sich dem klassischen Diskurs über das Exzerpieren (*De arte excerpendi*) zurechnen lassen.^36^ Der „Beweis“, dass es sich bei einem Exzerpt wirklich um eine philosophisch relevante Aussage mit apriorischem Wahrheitsbezug und eben nicht nur um eine bloße Textkopie handelt, dürfe nicht von äußerer „Autorität und gute[m] Glauben“ abhängen, sondern könne jenseits überkommener Kanonisierung nur „durch die Exposition ihres Wesens geliefert werden.“^37^

Die philosophische Wesenserkenntnis im Sinne Hegels, die das Motiv des „platonischen Pedantismus“ ersetzt, bildet für Marx folglich die Voraussetzung, um sich vor der bloß eklektischen Kopie zu hüten, die den „Strom“, wie es in seiner Kritik der Historischen Rechtsschule heißt, als Gegenstand rationaler Erkenntnis durch die bloße „Quelle“ ersetzt.^38^ Während der eklektische Historismus den philosophischen Wald vor lauter empirischen Bäumen nicht mehr zu sehen vermag, leidet die bloße Logifizierung der Philosophiegeschichte – mit Pierre Bourdieu formuliert – am Mangel der „Geschichtsvergessenheit“, weshalb Marx eine Synthese aus spekulativer Philosophie und materialer Textarbeit vorschlägt.^39^ Er kommt in seinen philosophiehistorischen Exzerpten also zu einer methodischen Reflexion über die Praxis der Exzerpierens, die den Rahmen des vormodernen gelehrten Buchwissens sprengt, aber den Diskurs wie die Praxis gleichzeitig fortschreibt.

Die von Marx aktualisierte Metapher des philosophischen Maulwurfs nimmt die vormoderne Problematik der primordialen *philosophia perennis* zwar auf, übersetzt die alte Vorstellung einer teleologischen und sich mehr oder weniger triumphalistisch offenbarenden göttlich‐absoluten Weisheit aber durch ein Wesen, das sich stumm und halbblind durch den kontingenten Untergrund des Geistes gräbt. Karlheinz Stierle hat darauf hingewiesen, dass diese Metapher literarisch vermittelt ist und auf die konkrete Suche nach wahrhaftiger Bedeutung in Texten und Bibliotheken verweist. Dennoch muss der philosophische Maulwurf vom exoterisch‐phänomenologischen Bewusstsein unterschieden werden. Schon bei Hegel steht der nahezu blinde Maulwurf für die „Arbeit des Geistes“, die äußerlich alles andere als zielstrebig erscheint, trotzdem aber „innerliches Fortarbeiten“ ist.^40^ Dies ist zwar immer noch eine Fortschrittsgeschichte, die aber dialektisch zugleich kontraproduktiv ist und gleichsam im untergründigen Wesen der Geschichte verborgen liegt. Für Stierle steht die Maulwurfs‐Metapher bei Hegel für einen „Weltgeist“, „der sich durch das Bestehende wühlt“ und folglich eine „Projektion des Denkers [ist], der sich durch die Sedimentierungen des Wissens wühlt und durch die Ordnung der Diskurse hindurch die lebendige Erfahrung ihres inneren Zusammenhangs sucht.“^41^ Demnach ist es keineswegs so, dass das Wühlen in der Literatur und in den Exzerpten der Wesenserkenntnis vollständig äußerlich wäre; der bibliophile Maulwurf gräbt sich vielmehr durch die Texte und kommt nur an bestimmten Momenten an die Oberfläche, wo er kurz in die platonische Sonne blinzelt. Erst von diesem Moment bestimmt sich nachträglich, was an der Lektüre wesentlich oder unwesentlich, esoterisch oder exoterisch war. Somit kann Stierles Interpretation erklären, warum das vom jungen Marx 1839/40 formulierte Programm einer historisch‐philosophischen Vermittlung für ihn noch mit dem hegelschen System kompatibel erscheinen konnte. Darüber hinaus wird deutlich, wie theoriegeleitet und problemorientiert das Marxsche Exzerpieren ist und die Konstitution von Quellen wie von Erkenntnissen berührt.

Die Spezifik von Marx’ programmatischem Vorgehen zeigt sich auch im dritten Exzerptheft, wo er von der frontalen und grundsätzlichen Polemik gegenüber Plutarch zu einer stärker über Inhalte geprägten Auseinandersetzungen übergeht. Obwohl die Kritik Plutarchs an der epikureischen Theologie mitunter gerechtfertigt ist, trägt er „in seiner Polemik gegen Epikur“ immer noch „die epikuräische Lehre vor“, „nur daß dieser einfach, abstrakt, wahr und dürr die Consequenzen entwickelt und weiß, was er sagt, während Plutarch überall etwas andres sagt, als er zu sagen meint, aber im Grund auch etwas andres meint, als er sagt.“^42^ Marx spricht Plutarch „alle philosophischen Fühlhörner“ ab und kritisiert einige „pinselhafte, des dümmsten Dorfschulmeisters“ würdige Äußerungen;^43^ danach wendet er sich im vierten Arbeitsheft Lukrez’ Lehrgedicht *De rerum natura* zu.^44^

Dieses Exzerpt ist ein Beispiel dafür, dass eine offenbar ohne große Erwartungen begonnene Lektüre („Es versteht sich, daß Lucretius nur wenig benuzt werden kann“^45^) dennoch enorm wichtig wird, so dass Marx während des Schreibens seine ursprüngliche Einschätzung ändert, den Autor in seinen persönlichen Kanon dauerhaft hoch einordnet. Marx wird nämlich schnell klar. „wie unendlich philosophischer Lucretius den Epikur auffaßt, als Plutarch.“^46^ Inhaltlich wird Marx von der Darstellung und Interpretation von Epikurs Atomtheorie bei Lukrez im Bann gehalten, die eine natürliche Alternative zur göttlichen Schöpfungslehre bietet. Das zuvor meist kursorische Exzerpt nimmt nun statarische Züge an, d. h. es folgt der Vorlage Zeile für Zeile. Im Mittelpunkt des Interesses steht die Idee einer autonomen Abweichung der Atome von einer ansonsten geraden Bewegungs‐ bzw. Falllinie („declinatio atomorum a via recta“).^47^ Die natürliche Abweichung des Atoms von seiner Bewegung bildet nicht nur die Voraussetzung für eine natürlich‐rationale Theorie der Freiheit und des Selbstbewusstseins; sie führt Marx auch zu der Einsicht, „warum Demokrits Lehre eine ganz verschiedne […] wie die epikureische war.“^48^ Mit dieser Feststellung hatte er beim Exzerpieren den Gegenstand seiner geplanten Dissertation gefunden, der dann auch in den Titel einfloss („Differenz der demokritischen und epikureischen Naturphilosophie“).^49^

Das Lukrez‐Exzerpt findet im fünften Heft eine Weiterführung, wobei Marx nach der Auswertung der Bücher IV und V einen mehrseitigen Exkurs einfügt, der sich in Form eines Fließtextes bemüht, die neuen Erkenntnisse über die epikureische Naturphilosophie mit dem hegelschen Konzept der Philosophiegeschichte zusammenzudenken. Ebenso wie bei der Bewegung des Atoms gebe es in Hegels Philosophie „Knotenpunkte“ bzw. „Knotenlinien“, die vom „Fortgang der graden Linie abbrechen“ und die „Fastnachtszeit der Philosophie“ repräsentieren.^50^ Im Kontext der Metapher des philosophischen Maulwurfs wären dies Momente, in denen dieser kurz in die Sonne blinzelt und in eine neue Richtung weitergräbt bzw. liest. In der Folge erarbeitet sich Marx eine Position, die die logisch vollendete Totalisierung der hegelschen Philosophie inklusive ihrer Abschließung „gegen die erscheinende Welt“ als notwendige Voraussetzung für die praktische Öffnung entwirft.^51^ Gleichzeitig mit dieser praxeologischen Deutung Hegels avanciert er vom *lector* zum *auctor*.^52^ „Titanenartig sind aber die Zeiten, die einer in sich totalen Philosophie und ihren subjektiven Entwicklungsformen folgen, denn Riesenhaft ist der Zwiespalt, der ihre Einheit ist.“^53^ – Diese emphatische Selbstverortung richtet sich gegen Epigonentum und die Annahme, die Zeiten ließen nichts anderes zu.

In den nächsten Exzerpten widmet sich Marx zunächst der neueren Literatur, die die Philosophie christlich‐neuplatonisch immer noch bzw. wieder als Apologie der Offenbarung behandelt. Bei dieser Wiederholung einer Problematik, die seit der Frühaufklärung in der Philosophiegeschichte immer wieder diskutiert wurde, geht es ihm nach eigenen Worten buchstäblich darum, „die subjektive Form der Platonischen Philosophie in einigen Zügen noch weiter [zu] bestimmen“.^54^ Dies impliziert die Frage, ob die platonische Philosophie bei der subjektiven Fortentwicklung des hegelschen Systems eine Rolle spielen kann? Dazu setzt Marx sich mit einem Text des Tübinger Theologen Ferdinand Christian Baur (1792–1860) auseinander, der sich beim Vergleich von Sokrates und Christus sowohl der hegelschen Philosophie als auch der von Friedrich Schleiermacher in die neutestamentliche Forschung eingeführten historisch‐kritischen Methoden bedient hatte.^55^ Findet Marx hierin noch eine interessante Auseinandersetzung mit dem Platonismus, die ihn zu kommentierenden Reflexionen über die Frage der Mythologie und des Enthusiasmus in der Philosophie anregen, kann er Ritters *Darstellung der antiken Philosophiegeschichte* wenig abgewinnen.^56^

Nachdem Marx den Rest des fünften Exzerptheftes mit einem Schema der hegelschen Naturphilosophie aus der *Enzyklopädie der philosophischen Wissenschaften* ausgefüllt hat,^57^ kehrt er im sechsten Heft wieder zu antiken Quellen zurück und fertigt kursorische Auszüge aus Seneca, Stobaios und Alexandrinus an.^58^ Seneca liest er in einer Werkausgabe von 1672 und exzerpiert kommentarlos alle Epikur betreffenden Stellen.^59^ Dabei hat er sich wohl am Register orientiert. Den bruchstückhaft überlieferten Text von Stobaios aus dem 5. Jh. sieht Marx in einer Edition von 1609 durch.^60^ Die von ihm in griechischer Sprache notierten Aussagen von und über Epikur überschneiden sich zum Teil mit dem Seneca‐Exzerpt. Auch hier hält er sich mit eigenen Kommentaren zurück. Von Clemens Alexandrinus, einem frühchristlichen Kirchenvater, der griechische Philosophie apologetisch mit christlicher Theologie verband, exzerpiert Marx auf wenigen Seiten dessen Hauptwerk, die Bücher *Stromateis* in einer Werkausgabe von 1688.^61^ Seine Aufmerksamkeit richtet sich auf die Verurteilung der epikureischen Philosophie durch den Apostel Paulus.^62^

Im siebenten Heft erkundet Marx zwei Schriften von Cicero,^63^ *De natura deorum* und *De finibus bonorum et malorum*. Als die wahrscheinlich von Marx benutzte Cicero‐Ausgabe gilt eine Werkausgabe von 1745, die sich in seinem persönlichen Besitz befand.^64^ Exzerpiert werden aus verschiedenen Kapiteln einzelne Epikur betreffende Sätze zunächst wiederum ohne jeglichen Kommentar. Marx schreibt aus den einschlägigen Kapiteln kursorisch einzelne Zitate heraus oder fasst mit eigenen Worten das jeweilige Kapitel in einem Absatz zusammen. Er kommt hier nochmals auf das Verhältnis von Logischem und Historischem und damit auf die grundlegende problemgeschichtliche Dimension aller Exzerpthefte zurück und notiert Ciceros Bewertung der Deklination der Atome.^65^

Die beschriebene Exzerpierpraxis zeigt nicht nur ein theoriegeleitetes Vorgehen an, das zur Reflexion des Umgangs mit Quellen wie deren akribischer Aneignung zwingt, sondern auch – an der Maulwurf‐Metapher exemplifiziert – eine Einbettung in Traditionen der Philosophiegeschichtsschreibung und deren Modifikation. Sie sind von einer sich abzeichnenden emphatischen Autorschaftsidee getragen. Diese Variante des Exzerpierens kann als Vorläufer des auf Disziplinen konzentrierten Exzerpierens gelten, das Marx später in einer Problemgeschichte der Politischen Ökonomie realisiert.

## 4 Der „Maulwurf“ als „Copist“: Das Gülich‐Exzerpt von 1846/47

Das umfangreiche Exzerpt aus Gustav von Gülichs *Geschichtliche Darstellung des Handels, der Gewerbe und des Ackerbaus der bedeutendsten handeltreibenden Staaten unsrer Zeit*,^66^ das Marx 1846/47 anlegt, unterscheidet sich hinsichtlich der disziplinären Textgattung erheblich von den bisher behandelten Konvoluten. Zudem setzt er sich hier, anstatt mit einem historischen Bündel von Primär‐ und Sekundärautoren, einzig mit dem Werk *eines* Autors auseinander, wobei es in dem Exzerpt nur um die präzise Erfassung wirtschaftshistorischer Daten geht. Das Gülich‐Exzerpt wurde im Rahmen dieser Untersuchung aus einer ganzen Reihe weiterer Manuskripte vor allem deshalb ausgewählt, weil es eine andere Form des Exzerpierens repräsentiert und bis dato zu wenig beachtet wurde. Zu klären ist, ob das andere Interesse und der andere Stoff sich auf die Formen und Praktiken des Exzerpierens auswirken. Gibt es trotz des Wechsels der Wissenschaftsdisziplin Kontinuitäten im problemgeschichtlichen Charakter des Exzerpierens? Wie gestaltet sich etwa die von Marx problematisierte Spannung von exzerpierter Kopie und innovativer Autorenlogik, zumal wenn es um die objektive Erfassung von wirtschaftsgeschichtlichen Daten geht? Verändert der Umgang mit statistischem Material die gelehrte Praxis des Exzerpierens? Und wie wirkt sich der Wechsel von der philologischen *Lese*perspektive zur sozialwissenschaftlichen Methode, die nicht allein mit Texten, sondern auch mit „undokumentierten“ sozialen Praktiken *in actu* umzugehen beansprucht, auf die Form des Exzerpierens aus? Hat all dies Konsequenzen für das Ordnungssystem und die Materialität des Repositoriums?

Marx hält, um mit dem letzten Punkt zu beginnen, am Exzerptheft als materialem Repositorium fest. Die Exzerpthefte werden speziell zur Erfassung der komplexen Ordnung der fünf Bände von Gülich angefertigt. Es handelt sich um drei großformatige Hefte, deren gefaltetes Format zwischen 334×211 und 318×205 Millimetern schwankt. Die beiden ersten Hefte enthalten 40 bzw. 44 Seiten und das dritte Heft umfasst 116 Seiten. In der MEGA^2^ sind sie in Band IV/6 ediert. Aus Marx’ Notizbuch geht hervor, dass er 1844 die ersten vier Gülich‐Bände erwarb und den letzten, 1845 erschienenen Band später nachkaufte.^67^ Die umfangreichen Marginalien (auf 106 Seiten im ersten und 192 Seiten im zweiten Band) belegen das intensive Lesestudium von Marx.^68^ Gerade die Tatsache, dass er das fünfbändige Werk selbst besaß und mit zahlreichen Marginalien bearbeitete, dennoch aber ausführlich exzerpierte, verweist auf die große Bedeutung des sorgfältig geplanten und systematisch sowie extensiv durchgeführten Exzerptes (in der MEGA‐Edition umfassen die drei Exzerpthefte insgesamt fast 1.000 Seiten!). Offensichtlich schätzte er das Werk Gülichs und legte das Exzerpt gezielt als Wissensspeicher für die internationale Wirtschaftsgeschichte an, um diesen für sein seit 1844 geplantes Großprojekt einer Kritik der politischen Ökonomie langfristig nutzen zu können.

Das Exzerpt entstand zwischen September 1846 und Dezember 1847 in Brüssel und schließt sich daher chronologisch und thematisch überlappend an jene zwischen Oktober 1845 und Mai 1847 angefertigten Manuskripte an, die später unter dem Titel *Die deutsche Ideologie* bekannt wurden. Insofern stehen die ausgedehnten historischen Studien des Exzerptes in einem direkten Zusammenhang mit der sozialwissenschaftlichen Revision der junghegelianischen Philosophie, mithin also mit der eigenen spekulativen Vergangenheit. Auch wenn zu diesem Zeitpunkt von einem fertigen Konzept eines historischen Materialismus noch keine Rede sein kann, so bedeutet die sozialwissenschaftliche Kritik der Philosophie dennoch eine massive Aufwertung der historischen Methode. Gleichzeitig tritt die Absicht hervor, das alte kontemplative Wissensmodell des philologisch‐philosophischen Lesers durch die sozialwissenschaftliche Perspektive des handelnden Akteurs zu ersetzen, was eine erkenntnistheoretische Umkehr des Subjekt‐Objekt‐Verhältnisses und einen Bruch mit dem philologischen Paradigma des Übersetzens bedeutet, in dem die Sprache nur in toter Form behandelt werden kann, um sie überhaupt entziffern zu können.^69^ Stattdessen geht es Marx nun um die „Sprache des wirklichen Lebens“.^70^

Trotz dieses Paradigmenwechsels hin zur Sozialwissenschaft bleibt die 1839/40 diagnostizierte problemgeschichtliche Dimension der Exzerptarbeit, die zwischen Kopie und wesentlichem Wissen unterscheidet, auch im Exzerpt von 1846/47 relevant. Wenngleich es nun um die lebendige Sprache des Sozialen inklusive der epistemologischen Umorientierung vom *lector* zum *auctor* geht, fungiert das klassische Exzerpt weiter als zentrales Mittel der Wissensaneignung. Marx sucht aber nicht mehr nach wörtlichen Zitaten – in der Tat wird kaum zitiert – und philosophisch passenden Interpretationen, sondern einzig und allein nach ökonomischen und historischen Fakten. Obwohl auch diese Variante des Exzerpierens problemgeschichtlichen Charakter trägt, hat sich dieser im Vergleich zu den philosophiehistorischen Heften verändert. Dies schlägt sich auch in der Form des Exzerpierens nieder. Das Wesentliche, nach dem der Maulwurf gräbt, ist jetzt die sachlich‐objektive Information (*data bruta*), die sich auf die anerkannte wissenschaftliche Autorität des Textes stützt. Im Vergleich der Exzerptformen fällt zuerst die im Gülich‐Exzerpt komplett fehlende polemische Streitlust auf. Marx eignet sich mit Hilfe von Gülich den neuesten Stand der modernen deutschsprachigen Wirtschaftsgeschichte an. Das Exzerpt ist nahezu von einem Lehrer‐Schüler‐Verhältnis geprägt, da statarisch Kapitel für Kapitel, Absatz für Absatz vorgegangen wird und nach der Lektüre eine am sachlich Wesentlichen orientierte Zusammenfassung folgt. Kursorisches und statarisches Exzerpieren gehen hierbei eine neue Verbindung unter Verzicht auf die exakte Formulierung ein.

Anders als zuvor bei Hegel speist sich die Autorität von Gülich für Marx nicht mehr aus dem normativen Bereich der Philosophie; Gülich gilt ihm als „ein aufrichtiger Philanthrop“, der „eine sehr wissenschaftliche Geschichte der Industrie und des Handels geschrieben“ habe.^71^ Dennoch bildet das universale Konzept der Weltgeschichte eine Brücke von den philosophie‐ zu den wirtschaftsgeschichtlichen Exzerpten. Brannte 1839/40 „der beseelende Spiritus Weltgeschichtlicher Entwicklungen“ für Marx im „reinen idealen Feuer der Wissenschaft“,^72^ so ist es 1846/47 die internationale Arbeitsteilung und die Erfindung der Industrie, die dem exzerpierenden Maulwurf im Manuskript zu Feuerbach als „weltgeschichtliche[s] Faktum“ als wesentlich gilt.^73^ Dass „Geschichte zur Weltgeschichte“ wird, „wenn in England eine Maschine erfunden wird, die in Indien & China zahllose Arbeiter außer Brot setzt & die ganze Existenzform dieser Reiche umwälzt“,^74^ hat er zuvor von Gülich exzerpiert.^75^ Dieses Beispiel steht nicht nur für die parallele Exzerpt‐ und Manuskriptproduktion; es zeigt auch an, wie das Konzept der Weltgeschichte in sozialwissenschaftlichen Exzerpten und Manuskripten die problemgeschichtliche Sicht anleitet.

Seinem aktuellen Interesse folgend, startet Marx das erste Exzerptheft in umgekehrter Reihenfolge zur Literaturvorlage mit dem fünften und letzten Band. Das Exzerpt berührt – ausgehend von allgemeinen Problemen des internationalen Handels – Fragen der Zirkulation von Gold und Silber, des Warenpreises und der Handelsbilanz, um dann zur Besteuerung, Bevölkerungsentwicklung und den kontemporären ökonomischen Theorien fortzuschreiten. Anschließend widmet sich Marx der konkreten Wirtschaftsgeschichte der einzelnen Staaten, die er mit Gülichs Darstellung der deutschen Länder im vierten Band beginnt. Diese wird in ihrer diachronen Entwicklung vom Mittelalter bis zur Gegenwart um 1840 nachvollzogen. Danach setzt das zweite Heft die Auswertung des vierten Bandes fort und schreitet von den deutschen Territorien über die Niederlande, Dänemark, Portugal, Spanien, Türkei und Griechenland nach Afrika und Asien (u. a. Birma, Japan, China) fort. Das zweite Exzerptheft resümiert die Entwicklung in Polen, Russland, der Schweiz und Österreich. Erst im dritten Arbeitsheft thematisiert Marx die Wirtschaftsgeschichte Großbritanniens, die bei Gülich den Anfang des ersten Bandes bildet. Marx folgt Gülich im Blick auf die britische Entwicklung sehr ausführlich vom ersten in den dritten Band, wo das koloniale Empire behandelt wird. Daran schließen Auszüge zu den Vereinigten Staaten und Frankreich an, wobei die Informationen nun quer aus verschiedenen Bänden (Band 1, 2, 3 und 5) zusammengetragen und systematisiert werden. Dann wendet sich das Exzerpt nochmals der Behandlung Deutschlands in Band 2 zu. Im abschließend als „Zusammenfassung“ überschriebenen Teil exzerpiert Marx die bei Gülich als Einleitung konzipierte allgemeine europäische Entwicklung des Handels von den Kreuzzügen bis zur Wirtschaftskrise von 1825.

Insgesamt lassen sich also fünf Phasen des Exzerpierens unterscheiden, die der disziplinären Sachlogik folgen:^76^ nach der ersten Phase, wo es um allgemeine wirtschaftspolitische Grundfragen geht, folgt in der zweiten Phase die Darstellung einzelner Staaten inklusive einzelner länderbezogener ökonomisch‐historischer „Rückblicke“. Die dritte Phase ist von durchgehend chronologischem Vorgehen geprägt, was dafür spricht, dass Marx die einzelstaatliche Historisierung nicht mehr genügte und die Wirtschaftsgeschichte nun in der komplexen Verschränkung des Staatensystems über den sich herausbildenden Weltmarkt erfasst werden sollte. Diese methodische Umorientierung fällt mit der Anlage eines dritten Exzerptheftes zusammen, das Marx beginnt, obwohl im zweiten Heft noch Platz gewesen wäre. In der vierten Phase ergänzt er die historische Betrachtung der einzelnen Länder durch die aktuelle Entwicklung, wie sie Gülich im letzten Band darstellt. Einige dieser Nachträge (u. a. zu Portugal, Spanien, Russland, Niederlande) werden nun in die noch leeren Seiten des zweiten Exzerptheftes eingetragen. In der letzten Phase wertet Marx das gesammelte Material aus, wobei er in einer zweiten Verarbeitungsstufe der Auszüge auf die tabellarische Form zurückgreift, die er schon in früheren Exzerpten verwendet hatte.^77^ Dabei wird über die synchronoptische Darstellung des exzerpierten Materials, die sowohl historisch als auch nach verschiedenen staatlichen Wirtschaftsakteuren und gelegentlich nach sachlichen Momenten (Ackerbau, Krieg und Konkurrenz, Kolonialhandel, Currency etc.) angelegt ist, „eine über ein reines Exzerpt hinausgehende Verarbeitungs‐ und Verallgemeinerungsstufe erreicht. Hier erfolgt durch Marx schon beim Exzerpieren eine Aufbereitung des Materials sowohl zur Selbstverständigung als auch für seine spätere Verwendung.“^78^ Das Gülich‐Exzerpt ist besonders extensiv, aber in dieser Hinsicht keine Ausnahme; es repräsentiert vielmehr als neue Variante ein Muster für andere an Fakten orientierte Exzerpte zur Geologie, Natur‐ und Technikwissenschaft.

## 5 Fazit

Das Exzerpieren bildet zweifellos die Basis von Marx’ wissenschaftlich‐literarischer Produktionsweise. Marx bleibt einer gelehrten Praxis treu, die im älteren Buchwissen des 17. und 18. Jahrhunderts wurzelt. In diesem Sinne verwendet er das klassische Exzerptheft als materielles Repositorium, das stets Ordnungsprinzipien folgt, die aus dem Untersuchungsgegenstand abgeleitet werden. Gleichwohl entwickelt Marx während der Exzerptarbeit ein problemgeschichtliches Sensorium für die methodische Spannung zwischen Repetition und Innovation, die er mit Hilfe des Konzepts der Weltgeschichte in das Verhältnis von Historischem und Logischem übersetzt. Da die Fetischisierung beider Methoden abgelehnt wird, sucht er beim Exzerpieren wie Publizieren nach einer alternativen Praxis, die über traditionelle Exzerpierpraktiken und den quellenkritischen Historismus, aber auch über die abstrakte Logifizierung der Geschichte hinausgeht. Die untersuchten Exzerpte lassen sich als reflektierten Versuch einer methodischen Synthese verstehen. Das disziplingeschichtlich übergreifende Konzept der Weltgeschichte, das metaphorisch im Bild eines bibliophilen Maulwurfs illustriert wird, fungiert als heuristisches Muster der Urteilskraft bei der empirischen Selektion und logischen Bewertung der Quellen. Im Zuge dieser Synthese verbinden sich quellenkritische, philologische Exaktheit – Marx versucht auf die original‐getreue Textpräsentation zurückzugreifen, was in der Verwendung historisch‐klassischer Editionen deutlich wird – und universalistischer Geschichtslogik.

Das Verhältnis von Lektüre und Empirie während des Exzerpierens ist vom disziplingeschichtlichen Charakter – im Untersuchungsfall von der Philosophie‐ bzw. Wirtschaftsgeschichte – abhängig. Wiewohl Marx zwischen den untersuchten Exzerpten im Zuge des Übergangs zur Sozialwissenschaft eine methodische Wende von der philologisch‐philosophischen zur sozialwissenschaftlichen Lektüre von Texten vornimmt, hält er prinzipiell an den traditionellen Formen für die Aufzeichnung von Exzerpten fest. Durch die flexible Handhabung seines problemgeschichtlichen Konzepts von Weltgeschichte besitzt er ein Ordnungsmuster, das beim kursorischen und statarischen Exzerpieren disziplinübergreifend verwendet werden kann. Die gedrängte Darstellung der im Gülich‐Exzerpt verarbeiteten Fakten kann als eine Art Speicher verstanden werden, der das gesammelte Material und Wissen für zukünftige Arbeiten verfügbar hält. Dieses Exzerpt markiert den Anfang einer Praxis des Exzerpierens, die in ihrer Intensität geradezu den Eindruck einer förmlichen Inkorporation von Wissen erweckt. Es ist trotz beobachtbarer Ansätze zu einer sozialökonomischen Konzeption von (Welt‐)Geschichte hinsichtlich der eingesetzten Exzerpiermethoden weniger reflektiert als die frühere Auseinandersetzung mit der Philosophiegeschichte in Form des Exzerpierens antiker Autoren. Dass die eher theoretisch erfolgende Ausbildung der materialistischen Auffassung der Geschichte, die sich in den Manuskripten zur *Deutschen Ideologie* abzeichnet, parallel dazu im Ringen mit Bergen von empirischem Material in den Gülich‐Exzerpten erfolgt, erlaubt, wenn die Exzerpiermethoden und umfangreichen Exzerpte berücksichtigt werden, viele gängige Darstellungen des Marxschen Denkweges infrage zu stellen.

## Footnotes

1 Marx 1975b, S. 191.

2 Das macht die Marx‐Exzerpte im Untersuchungszusammenhang des internationalen Forschungsnetzwerkes, das von Elisabeth Décultot an der Martin‐Luther‐Universität Halle‐Wittenberg gebaut wurde, so interessant. Zum Forschungsprogramm siehe Décultot und Zedelmaier 2017.

3 Siehe hierzu zuletzt Heinrich 2018, S. 182–192 sowie Stedman Jones 2017, S. 82–88.

4 Die Rede vom Exzerpieren als Marx’ „Produktionsweise“ ist von Mohl 1993 eher aphoristisch geprägt worden. Wir haben sie für unser Forschungsvorhaben übernommen und inhaltlich‐programmatisch ausgeweitet: Bluhm und Rüdiger 2018.

5 Zum Kontext siehe Heinrich 2018, S. 331–339 sowie Stedman Jones 2017, S. 102–107.

6 Über Marx’ Verhältnis zur Antike informieren Haug 2001; Kondylis 1987; Nippel 2013 und speziell zu Aristoteles McCarthy 1992; Pike 1999 sowie Seidel 1979.

7 Siehe hierzu die Zeugenbeschreibung in Marx und Engels 1976, S. 567–575.

8 Marx und Engels 1976, S. 5–141, 147–151.

9 Die erste einschlägige Sammlung der Epikur‐Fragmente („Epicurea“) wurde erst 1887 von Hermann Usener vorgelegt. Wir verdanken diesen Hinweis Wilfried Nippel.

10 Schmidt‐Biggemann und Stammen 1998.

11 Hegel 1993, S. 222: „(D)as Studium der Geschichte der Philosophie ist Studium der Philosophie selbst, und zwar vornehmlich des Logischen.“ Siehe auch Kant 1942, S. 341.

12 Gassendi 1649. Zum eklektischen Konzept der frühneuzeitlichen Philosophiegeschichte siehe Albrecht 1994; Braun 1990 sowie Schneider 1998.

13 Klassisch hierfür ist das Werk von Jacob Brucker. Siehe dazu Schmidt‐Biggemann und Stammen 1998. Zur Entwicklung des Konzepts der *historia literaria* siehe insbes. Zedelmaier 1992 sowie Grunert und Vollhardt 2007.

14 Marx 1976a, S. 17.

15 Marx 1976a, S. 19.

16 Marx 1976a, S. 31f.

17 Noch im *Kapital* bezieht sich Marx auf dieses epikureische Konzept; Marx 1983b, S. 49.

18 Marx 1976a, S. 32–38. Exzerpiert wird aus Empiricus 1621.

19 Marx 1976a, S. 32.

20 Marx 1976a, S. 36f.

21 Ebenda.

22 Die von Marx verwendet Ausgabe wird von der MEGA^2^ als Plutarchus 1599 rekonstruiert.

23 Marx 1976a, S. 39–74.

24 Marx 1976a, S. 39. So ignoriere Plutarch vollständig, dass die epikureische Lust („voluptas“) eine Lust des Geistes („voluptas animi“) sei und das „höchste Gut des Epikurs“ in der „άταραξíα“ bestehe; ebenda.

25 Ebenda.

26 Bunia et al. 2011.

27 Marx 1976a, S. 64, 52, 54, 69.

28 Marx 1976a, S. 69.

29 Zum Gegensatz von Eklektizismus und Platonismus in der frühneuzeitlichen Philosophiegeschichte siehe Lehmann‐Brauns 2004.

30 Marx 1976a, S. 40.

31 Schmidt‐Biggemann 1998, S. 61. Auch bei Hegel ist die Philosophie noch „Rechtfertigung der Vorsehung“; Hegel 1986a, S. 493.

32 Marx 1976a, S. 39.

33 Marx 1976a, S. 137. Marx liest hier Hegels Bemerkungen aus den *Vorlesungen über die Geschichte der Philosophie* und der *Phänomenologie* zusammen: Hegel 1986c, S. 456 und Hegel 1986b, S. 403f.

34 Marx 1976a, S. 137.

35 Ebenda.

36 Siehe Cevolini 2006.

37 Marx 1976a, S. 137.

38 Marx 1975b, S. 191.

39 Damit befindet sich Marx durchaus im programmatischen Rahmen der von Bourdieu 2001, S. 62 unter Rekurs auf Spinoza vorgeschlagenen „wirklichen Wissenschaft der Kulturprodukte“. Er favorisiert nämlich gegenüber Platon die philosophische Traditionslinie von Aristoteles, Spinoza und Hegel; Marx 1976a, S. 104.

40 Hegel 1986c, S. 456.

41 Stierle 1984, S. 128.

42 Marx 1976a, S. 61, 63.

43 Marx 1976a, S. 72.

44 Carus 1801.

45 Marx 1976a, S. 74.

46 Marx 1976a, S. 79.

47 Marx 1976a, S. 84.

48 Marx 1976a, S. 85.

49 Marx 1975a.

50 Marx 1976a, S. 99.

51 Ebenda.

52 Zur epistemologischen Problematik von Lektoren‐ und Autorenperspektive in der Sozialwissenschaft siehe Bourdieu 2001, S. 62.

53 Marx 1976a, S. 101.

54 Marx 1976a, S. 102.

55 Baur 1837.

56 Ritter 1829.

57 Hegel 1830.

58 Marx 1976a, S. 120–130.

59 Seneca 1672.

60 Stobaeus 1609.

61 Alexandrinus 1688.

62 Siehe Marx 1976a, S. 129f.

63 Marx 1976a, S. 131–141.

64 Cicero 1745.

65 Marx 1976a, S. 132.

66 Gülich 1830–1845.

67 Marx 1983b, S. 989.

68 Ulrich Pagel verdanken wir die Auskunft, dass die Gülich‐Bände aus der Bibliothek von Marx mitsamt den Marginalien überliefert sind. Letztere sind einsehbar in Harstick et al. 1999, Nr. 517.

69 Hierzu Bourdieu 2001, S. 78.

70 Marx 2017, S. 135.

71 Marx 1983a, S. 296.

72 Marx 1976a, S. 104.

73 Marx 2017, S. 41f.

74 Marx 2017, S. 41.

75 Marx 1983b, S. 331.

76 Thümmler 2016, S. 218–221.

77 Marx 1983b, S. 891–937.

78 Thümmler 2016, S. 221.
